# Supplementary material for: Identification of photoperiod-induced specific miRNAs in the adrenal glands of Sunite sheep (Ovis aries)
Source: Front Vet Sci. 2022 Jul 22;9:888207. doi: 10.3389/fvets.2022.888207 (PMC9354845; doi:10.3389/fvets.2022.888207)
Supplement: Supplementary file 1 [file Data_Sheet_1.ZIP › Supplementary file/miRDeep2 file.zip!/miRDeep2 file/Novel_Structure/SP42A2/Novel_452.pdf]

Provisional ID	:	NC_019484.2_42105
Score total	:	2.7
Score for star read(s)	:	-1.3
Score for read counts	:	0
Score for mfe	:	2.4
Score for randfold	:	1.6
Score for cons. seed	:	
Total read count	:	61
Mature read count	:	61
Loop read count	:	0
Star read count	:	0

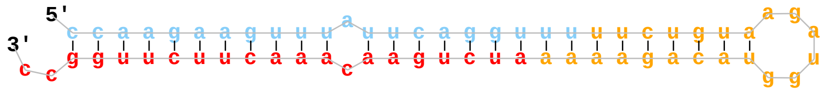

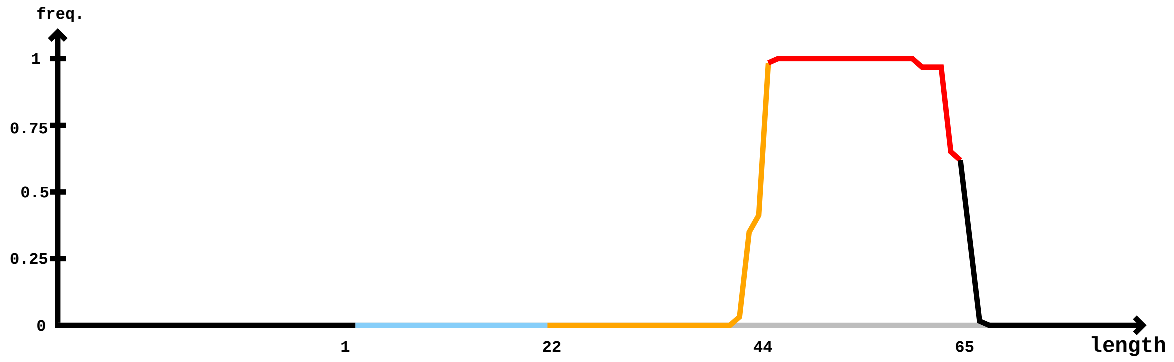

Star Mature

```
5'- uucaucuguaauccaauauacuggguuggccaagaaguuuauucagguuuuucuguaagaugguacagaaaaaucugaacaaacuucuuggccaacccaauacuuaauucu   -3'    exp
    .....................(((((((((((((((((((.((((((((((((((((......)))))))))))))))).))))))))))))))))))).............    reads    mm    sample
    .....................................................................aaaaucugaacaaacuuUu.......................    2        1     seq
    ......................................................................aaaucugaacaaacuucuugg....................    1        0     seq
    ......................................................................aaaucugaacaaacuuUuugg....................    19       1     seq
    .......................................................................aaucugaacaaacuuUuuggc...................    2        1     seq
    .......................................................................aaucugaacaaacuuUuuggcc..................    2        1     seq
    ........................................................................aucugaacaaacuuUuuggcc..................    25       1     seq
    ........................................................................aucugaacaaacuuUuuggcca.................    11       1     seq
    .........................................................................ucugaacaaacuuUuuggccaa................    1        1     seq
```
